# Supplementary material for: Bicontinuous Cubic Liquid Crystals as Potential Matrices for Non-Invasive Topical Sampling of Low-Molecular-Weight Biomarkers
Source: Pharmaceutics. 2023 Jul 28;15(8):2031. doi: 10.3390/pharmaceutics15082031 (PMC10459996; doi:10.3390/pharmaceutics15082031)
Supplement: Supplementary file 1 [file pharmaceutics-15-02031-s001.zip › pharmaceutics-2457564-supplementary.pdf]

Supplementary information

Bicontinuous Cubic Liquid Crystals as Potential Matrices for Non-Invasive Topical Sampling of Low-Molecular-Weight Biomarkers

Maxim Morin [1,2], Sebastian Björklund [1,2], Emelie J. Nilsson [1,2] and Johan Engblom [1,2,*]

1 Biofilms—Research Center for Biointerfaces, Malmö University, SE-205 06 Malmö, Sweden; maxim.morin@mau.se (M.M.); sebastian.bjorklund@mau.se (S.B.); emelie.nilsson@mau.se (E.J.N.)
2 Department of Biomedical Science, Faculty of Health and Society, Malmö University, SE-205 06 Malmö, Sweden
* Correspondence: johan.engblom@mau.se; Tel.: +46-70-608-7525

S1. Determination of the lattice parameter *a*

The positions of the Bragg peaks (in *q*) in the measured diffractograms are used to assign a liquid crystalline phase to each sample. The ratio between the Bragg peaks is determined by the conditions providing constructive and destructive interference, often described using Miller indices (*hkl*), and is henceforth unique for each liquid crystalline phase (space group) [1]. The ratio, derived from the Miller indices, between Bragg peaks of bicontinuous cubic liquid crystalline phases investigated here are as follows:

Bicontinuous cubic primitive phase (Im3m): √2: √4: √6: √8: √10: √12: √14: √16...,
Bicontinuous cubic diamond phase (Pn3m): √2: √3: √4: √6: √8: √9: √10: √11...,
Bicontinuous cubic gyroid phase (Ia3d): √6: √8: √14: √16: √20: √22: √24: √26....

This in combination with the lattice spacing *d*, where $|q| = 2\pi/d$, is used to determine the liquid crystalline phase's lattice parameter, *a*, which is the smallest repeat distance in the structure's unit cell, [1,2] using the following equation for cubic structures:

$$\frac{1}{d^2} = \frac{h^2 + k^2 + l^2}{a^2}$$

The lattice parameter *a* was determined using 3-6 Bragg peaks for each sample.

S2. Method validation for HPLC-UV

Table S1. LOD and LOQ for Trp and Kyn. LOD is defined as 3.3*σ/Slope, and LOQ is defined as 10*σ/Slope, where σ is the standard error of the intercept.

	Trp (μM)	Kyn (μM)
LOD	0.14	0.23
LOQ	0.43	0.69

Table S2. Precision of quantification of the analytical method. Calibration solutions were prepared for Trp and Kyn. The analyte peak areas were determined over three different days. The coefficient of variation is defined as CV = SD/Mean x 100%, the precision is defined as 100% - CV.

C (μM)	Mean (a.u.)		SD (a.u.)		CV (%)		Precision (%)	
	Trp	Kyn	Trp	Kyn	Trp	Kyn	Trp	Kyn
3.125	35.9	28.9	1.0	0.4	2.7	1.3	97.3	98.7
6.25	71.6	55.8	1.2	1.0	1.7	1.8	98.3	98.2
12.5	141.8	111.0	1.6	1.8	1.1	1.6	98.9	98.4
25	282.9	220.7	4.3	3.6	1.5	1.6	98.5	98.4
50	562.0	439.4	9.1	8.2	1.6	1.9	98.4	98.1
100	1120.7	876.1	18.8	14.1	1.7	1.6	98.3	98.4

Table S3. Accuracy of the analytical method. Calibration solutions were prepared for Trp and Kyn. The analyte concentrations were determined over three different days. The coefficient of variation is defined as CV = SD/Mean x 100%, the accuracy is defined as 100% - CV.

C (μM)	Mean (μM)		SD (μM)		CV (%)		Accuracy (%)	
	Trp	Kyn	Trp	Kyn	Trp	Kyn	Trp	Kyn
3.125	3.0	3.1	0.1	0.1	3.9	2.2	96.1	97.8
6.25	6.2	6.2	0.1	0.1	1.5	1.5	98.5	98.5
12.5	12.5	12.5	0.2	0.0	1.3	0.2	98.7	99.8
25	25.1	25.0	0.1	0.1	0.3	0.2	99.7	99.8
50	50.0	50.0	0.4	0.2	0.7	0.3	99.3	99.7
100	100.0	100.0	0.2	0.1	0.2	0.1	99.8	99.9

S3. The effect of the cationic lipid DOTAP on the phase behavior of GMO-water

The binary phase diagram of GMO-water has been well investigated in the broad range of temperatures (20-120 °C) and water content [3-6]. It is known that depending on temperature and hydration level, GMO can form a lamellar phase (Lα), two bicontinuous cubic phases: the gyroid (Ia3d) phase and the double diamond (Pn3m) phase, a hexagonal phase (H_{II}) and a fluid isotropic (L_2) phase. One aim of this work was to investigate how the addition of a cationic lipid DOTAP will affect the phase behaviour of GMO. DOTAP forms a lamellar phase when hydrated, which swells up to a final lattice parameter of around 708 Å. Further addition of water leads to a coexistence between fully swollen unilamellar vesicles and excess of water. SAXD results for three different concentrations of DOTAP (1.25, 2.5 and 5 wt %) in water are shown in **Figure S1**.

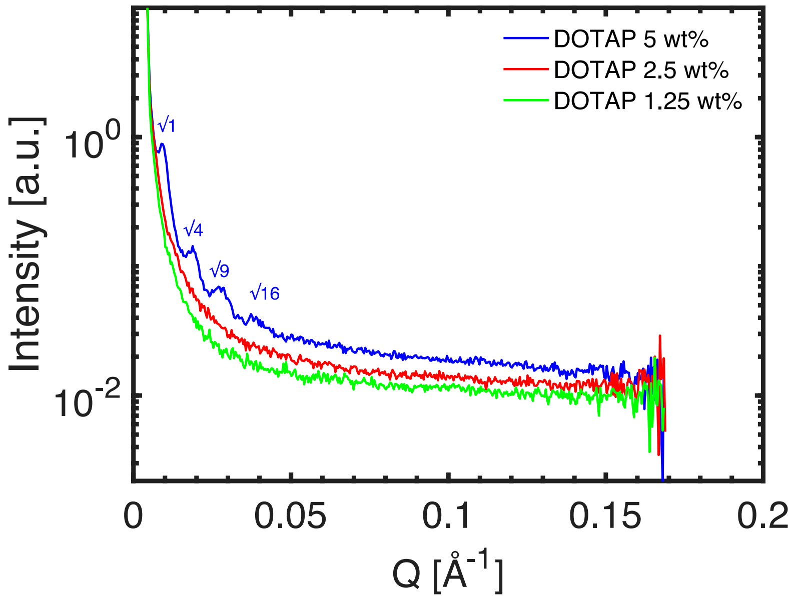

Figure S1. Diffraction patterns of DOTAP 5 wt % (blue), 2.5 wt % (red) and 1.25 wt % (green) in H_2O.

In total five series of samples containing GMO and DOTAP at ratios 97.5:2.5, 95:5, 90:10, 85:15 and 80:20 % (w/w) respectively were prepared, in which the water content was varied from 15 to 99 % (w/w) keeping the lipid ratios constant. The diffraction patterns for GMO with different compositions of DOTAP and water obtained from SAXD measurements at 25 °C are shown in **Figure S2**. Pure GMO at 15 wt % H_2O forms a lamellar phase (Lα), which goes over to a bicontinuous cubic phase Ia3d observed at 30 wt % H_2O. Higher amount of water added to GMO causes a phase transition into the Pn3m phase, which then coexists with excess of water (> 40 wt % H_2O). Addition of a small amount of cationic lipid DOTAP (2.5 wt %) to GMO, results in the formation of Ia3d phase already at 15 wt % of H_2O, which then swells up 30 wt % of H_2O. The presence of DOTAP (even at small quantities) causes the formation of a third bicontinuous cubic phase, which is not observed in case of pure GMO, namely, Im3m phase. Increase in water content leads to a substantial swelling of Im3m phase, which eventually changes to a lamellar phase (in samples with DOTAP ≥ 10 wt %). Interestingly that at DOTAP content up to 5 wt % of total lipid content increase in the water above 90 wt % resulted in the decrease of the lattice parameter, which could be due to the escape of DOTAP into the aqueous solution resulting in a decreased charge of the lipid bilayer.

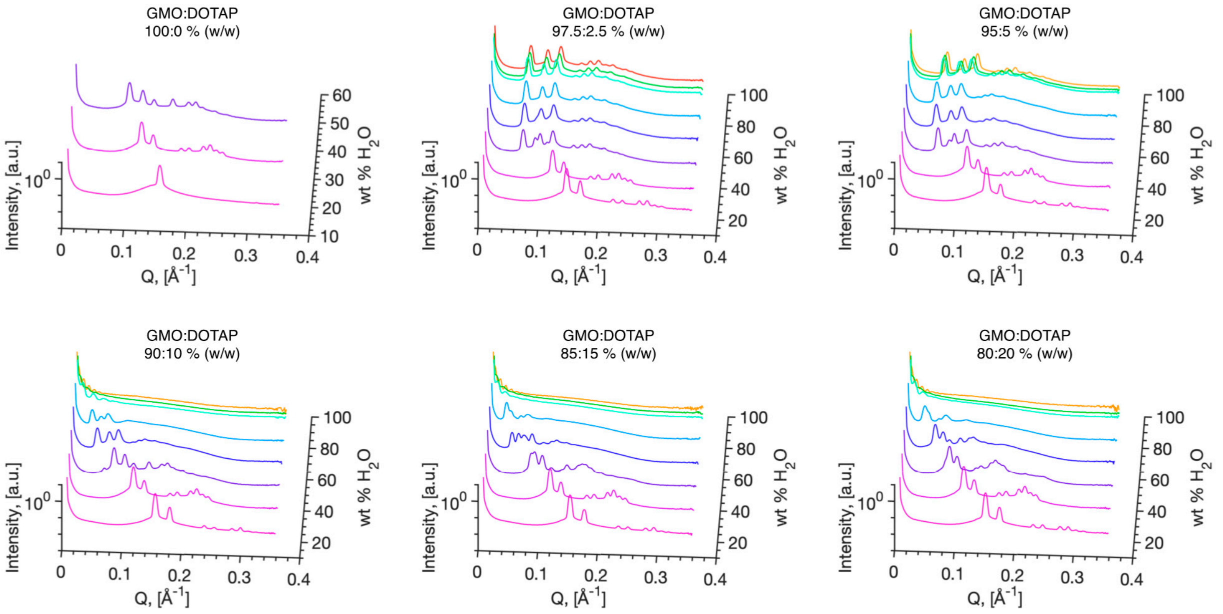

Figure S2. Electrostatic swelling of GMO in water caused by addition of different amounts of cationic lipid DOTAP at 25 °C. For pure GMO data shows diffraction patterns obtained from 15, 30 and 45 wt % H_2O, which represent a Lα phase, Ia3d phase and Pn3m phase. Above 40 wt % H_2O Pn3m phase coexists with the excess of water. Addition of DOTAP (< 5 wt %) to GMO results in a substantial swelling and formation of an additional bicontinuous cubic phase Im3m. Higher amount of DOTAP (between 10 and 20 wt %) results in even stronger swelling and phase transition into Lα phase at water contents above 90 wt %.

From the lattice parameter, a, it is possible to determine the radius of water channels in the cubic phase based on the minimal surface approach [7]:

$$r = \left(-\frac{\sigma}{2\pi\chi}\right)^{1/2} a - l$$

where l is the monolayer thickness, which can be determined accordingly:

$$\phi_l = 2\sigma\left(\frac{l}{a}\right) + \frac{4}{3}\pi\chi\left(\frac{l}{a}\right)^3$$

where $\boldsymbol{\chi}$ is the Euler-Poincaré characteristic and σ is the ratio of the minimal surface area in a unit cell to the quantity (unit cell volume)$^{2/3}$, which for $\boldsymbol{\chi}^{Pn3m} = -2$, $\sigma^{Pn3m} = 1.919$; $\boldsymbol{\chi}^{Im3m} = -4$, $\sigma^{Im3m} = 2.345$; and $\boldsymbol{\chi}^{Ia3d} = -8$, $\sigma^{Ia3d} = 3.091$ respectively. The radius of the water channel for the three cubic phases are:

For Ia3d: $r = 0.248 \cdot a - l$

For Pn3m: $r = 0.391 \cdot a - l$

For Im3m: $r = 0.306 \cdot a - l$

The average radii of curvature <R> can be calculated from following:

$$\langle R\rangle = \left(\frac{-Ha^3}{2\pi\chi}\right)^{1/3}$$

Where *H* is the "homogeneity index", $H^{Pn3m} = 0.7498$, $H^{Im3m} = 0.7163$ and $H^{Ia3d} = 0.7665$ (Engblom, Hyde, [7]). The minimal surface area per unit cell, A_{UC}, and the water channel length, L_w, can then be determined from the average radii of curvature as follows:

$$A_{UC} = -2\pi\chi\langle R\rangle^2$$

$$L_w = -\chi\langle R\rangle$$

Finally, it is possible to determine the volume fraction of the lipid from the average radii of curvature as:

$$\Phi_{lipid} = 1 - \Phi_{H_2O} = \frac{\left(3\left(\frac{\langle R\rangle}{l}\right)^2 - 1\right)}{2\left(\frac{\langle R\rangle}{l}\right)^3}$$

These equations were used to calculate the following:

Table S4. GMO:DOTAP 97.5:2.5 % (w/w).

**Lipid (wt %)**	**H_2O (wt %)**	**a (Å)**	**a/l**	**r (Å)**	**<R> (Å)**	**A_{UC} (Å^2)**	**L_w (Å)**	**Φ lipid**	**Φ H_2O**	**Symmetry**
84.480	15.520	105.300	6.194	8.860	26.112	34273.211	208.897	0.839	0.161	Ia3d
68.991	31.009	127.868	7.522	14.711	31.709	50538.645	253.669	0.727	0.273	Ia3d
54.623	45.377	101.424	5.966	22.657	39.633	19738.582	79.265	0.604	0.396	Pn3m
54.623	45.377	132.623	7.801	23.583	40.511	41246.147	162.044	0.593	0.407	Im3m
39.502	60.498	100.009	5.883	22.103	39.080	19191.479	78.159	0.611	0.389	Pn3m
39.502	60.498	135.972	7.998	24.607	41.534	43355.261	166.135	0.580	0.420	Im3m
24.899	75.101	136.830	8.049	24.870	41.796	43904.438	167.184	0.576	0.424	Im3m
10.097	89.903	138.602	8.153	25.412	42.337	45048.614	169.348	0.570	0.430	Im3m
7.594	92.406	133.440	7.849	23.833	40.760	41755.622	163.041	0.589	0.411	Im3m
2.501	97.499	130.233	7.661	22.851	39.781	39772.441	159.122	0.602	0.398	Im3m

Table S5. GMO:DOTAP 95:5 % (w/w).

Lipid (wt %)	H2O (wt %)	a (Å)	a/l	r (Å)	<R> (Å)	A_{UC} (Å^2)	L_w (Å)	Φ lipid	Φ H_2O	Symmetry
84.950	15.050	101.360	5.962	8.137	25.135	31756.328	201.081	0.860	0.140	Ia3d
68.780	31.220	133.595	7.859	16.132	33.129	55167.026	265.030	0.715	0.285	Ia3d
53.234	46.766	106.429	6.261	24.614	41.588	21734.737	83.177	0.579	0.421	Pn3m
53.234	46.766	138.602	8.153	25.412	42.337	45048.614	169.348	0.570	0.430	Im3m
39.379	60.621	155.618	9.154	30.619	47.535	56788.549	190.139	0.514	0.486	Im3m
25.380	74.620	160.248	9.426	32.036	48.949	60218.493	195.797	0.500	0.500	Im3m
10.023	89.977	141.336	8.314	26.249	43.172	46843.147	172.688	0.560	0.440	Im3m
7.425	92.575	135.972	7.998	24.607	41.534	43355.261	166.135	0.580	0.420	Im3m
4.971	95.029	129.436	7.614	22.607	39.537	39287.275	158.149	0.605	0.395	Im3m

Table S6. GMO:DOTAP 90:10 % (w/w).

Lipid (wt %)	H2O (wt %)	a (Å)	a/l	r (Å)	<R> (Å)	A_{UC} (Å^2)	L_w (Å)	Φ lipid	Φ H_2O	Symmetry
84.329	15.671	99.939	5.879	7.785	24.783	30872.346	198.262	0.876	0.124	Ia3d
70.009	29.991	134.064	7.886	16.248	33.245	55555.271	265.961	0.700	0.300	Ia3d
54.200	45.800	111.378	6.552	26.549	43.522	23803.195	87.045	0.556	0.444	Pn3m
54.200	45.800	141.336	8.314	26.249	43.172	46843.147	172.688	0.560	0.440	Im3m
40.026	59.974	186.60	10.976	40.099	56.998	81649.627	227.991	0.434	0.566	Im3m
25.072	74.928	289.250	17.015	71.511	88.354	196196.187	353.415	0.285	0.715	Im3m
9.983	90.017	301.212	17.718	75.171	92.008	212759.497	368.031	0.274	0.726	Im3m
9.983	90.017	406.416	23.907							Lα
7.020	92.980	534.739	31.455							Lα
7.020	92.980	402.800	23.694	106.257	123.039	380471.359	492.154	0.206	0.794	Im3m
4.972	95.028	708.843	41.697							Lα

Table S7. GMO:DOTAP 85:15 % (w/w).

Lipid (wt %)	H2O (wt %)	a (Å)	a/l	r (Å)	<R> (Å)	AUC ($Å^2$)	Lw (Å)	Φ lipid	Φ H_2O	Symmetry
84.840	15.160	101.360	5.962	8.137	25.135	31756.328	201.081	0.860	0.140	Ia3d
69.659	30.341	133.595	7.859	16.132	33.129	55167.026	265.030	0.702	0.298	Ia3d
54.983	45.017	178.173	10.481	27.187	44.183	98125.672	353.465	0.549	0.451	Ia3d
54.983	45.017	111.954	6.586	26.774	43.747	24049.749	87.494	0.554	0.446	Pn3m
39.761	60.239	156.743	9.220	44.287	61.249	47142.260	122.498	0.406	0.594	Pn3m
39.761	60.239	195.034	11.473	42.681	59.575	89200.144	238.299	0.416	0.584	Im3m
24.796	75.204	223.317	13.136	70.317	87.264	95691.956	174.527	0.289	0.711	Pn3m
24.796	75.204	271.073	15.945	65.948	82.801	172311.827	331.206	0.304	0.696	Im3m
9.880	90.120	324.061	19.062	82.163	98.987	246262.387	395.949	0.255	0.745	Im3m
9.880	90.120	454.974	26.763							Lα
7.568	92.432	402.800	23.694	106.257	123.039	380471.359	492.154	0.206	0.794	Im3m
7.568	92.432	534.739	31.455							Lα
5.039	94.961	708.843	41.697							Lα

Table S8. GMO:DOTAP 80:20 % (w/w).

Lipid (wt %)	H2O (wt %)	a (Å)	a/l	r (Å)	<R> (Å)	AUC ($Å^2$)	Lw (Å)	Φ lipid	Φ H_2O	Symmetry
85.413	14.587	102.947	6.056	8.531	25.529	32758.700	204.229	0.851	0.149	Ia3d
69.549	30.451	139.030	8.178	17.479	34.476	59746.684	275.811	0.680	0.320	Ia3d
54.581	45.419	184.341	10.844	28.716	45.713	105036.471	365.700	0.532	0.468	Ia3d
54.581	45.419	114.330	6.725	27.703	44.676	25081.744	89.352	0.543	0.457	Pn3m
40.044	59.956	156.743	9.220	44.287	61.249	47142.260	122.498	0.406	0.594	Pn3m
24.927	75.073	214.425	12.613	66.840	83.789	88223.405	167.578	0.300	0.700	Pn3m
24.927	75.073	261.269	15.369	62.948	79.807	160073.604	319.227	0.315	0.685	Im3m
10.016	89.984	532.082	31.299	145.817	162.529	663895.988	650.115	0.156	0.844	Im3m
10.016	89.984	417.487	24.558							Lα
7.505	92.495	663.117	39.007	185.914	202.554	1031153.665	810.218	0.126	0.874	Im3m
7.505	92.495	516.709	30.395							Lα
4.996	95.004	708.843	41.697							Lα

The effect of DOTAP on the swelling is shown in **Figure S3**, data used for these results is presented in **Tables S4-S8**.

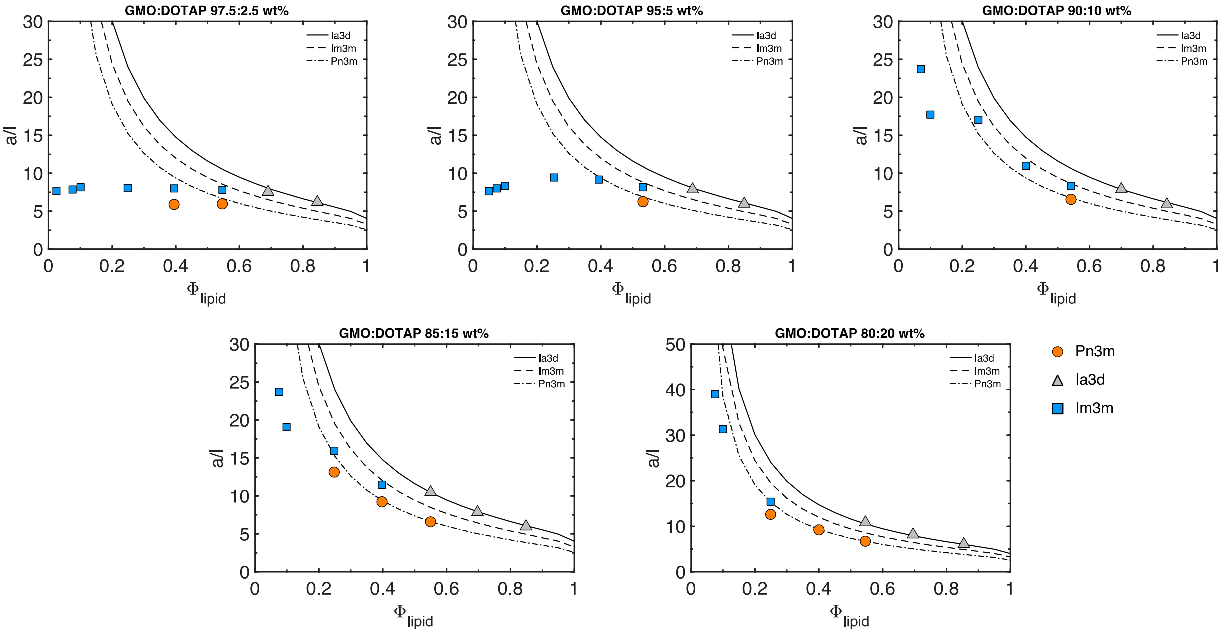

Figure S3. Swelling curves showing the ratio between the lattice parameter, a, and the lipid monolayer thickness, l, plotted as a function of the lipid weight fraction. The monolayer thickness for monoolein was used (17 Å).

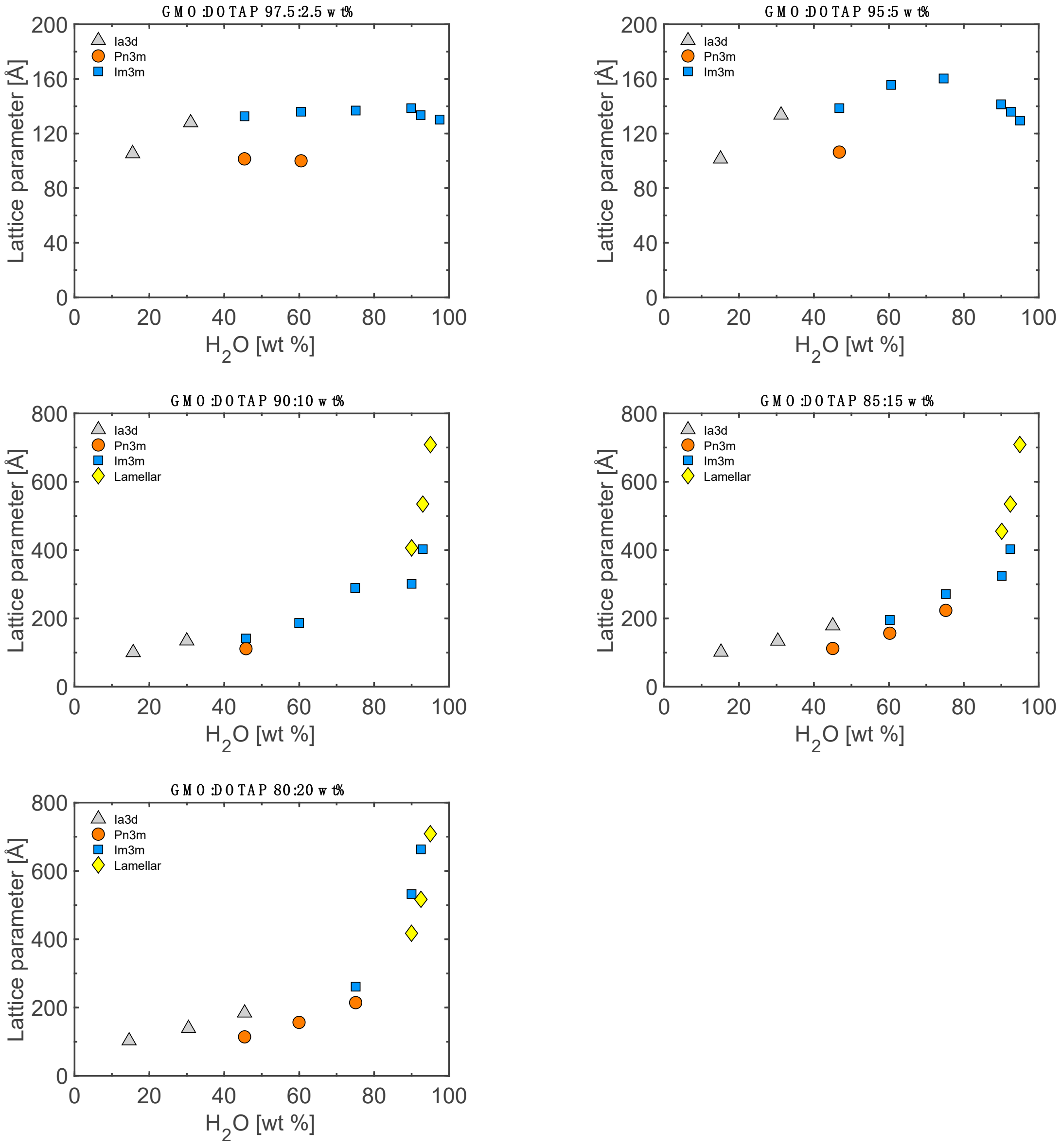

Figure S4. The change in the lattice parameter as function of DOTAP composition ranging from 2.5 wt % to 20 wt % of the total lipid content at various water contents.

S4. The effect of counter ions on the electrostatic swelling

The electrostatic swelling is strongly supressed by the Cl^- counterions. Im3m is not observed in any composition of GMO:DOTAP:H_2O.

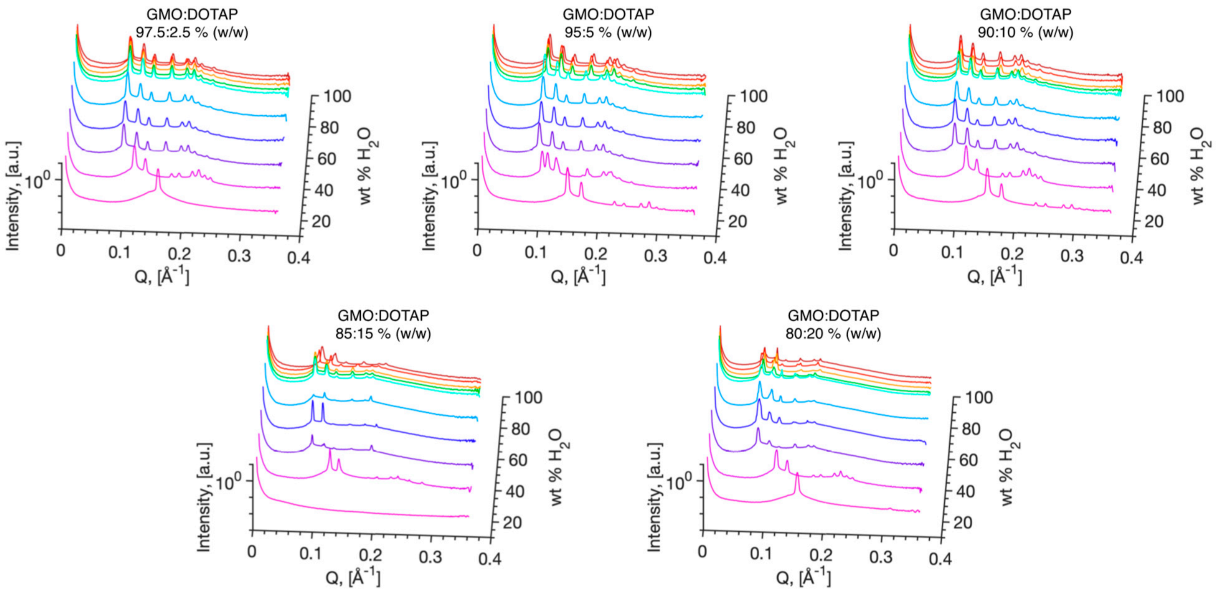

Figure S5. The effect of NaCl (150 mM) on the electrostatic swelling of GMO with various amounts of DOTAP.

References

1. Smart, L.E.; Moore, E.A. *Solid State Chemistry: An Introduction*, Third Edition (3rd ed.) ed.; CRC Press: 2005.
2. Seddon, J.M.; Templer, R.H. Chapter 3 - Polymorphism of Lipid-Water Systems. In *Handbook of Biological Physics*, Lipowsky, R., Sackmann, E., Eds.; North-Holland: 1995; Volume 1, pp. 97-160.
3. Larsson, K. 2 Cubic Phases in Monoolein Water-System. *Nature* **1983**, *304*, 664-664, doi:DOI 10.1038/304664c0.
4. Hyde, S.T.; Andersson, S.; Ericsson, B.; Larsson, K. A Cubic Structure Consisting of a Lipid Bilayer Forming an Infinite Periodic Minimum Surface of the Gyroid Type in the Glycerolmonooleate-Water System. *Zeitschrift Fur Kristallographie* **1984**, *168*, 213-219, doi:DOI 10.1524/zkri.1984.168.1-4.213.
5. Briggs, J.; Chung, H.; Caffrey, M. The temperature-composition phase diagram and mesophase structure characterization of the monoolein/water system. *J. Phys. II* **1996**, *6*, 723-751.
6. Qiu, H.; Caffrey, M. The phase diagram of the monoolein/water system: metastability and equilibrium aspects. *Biomaterials* **2000**, *21*, 223-234, doi:Doi 10.1016/S0142-9612(99)00126-X.
7. Engblom, J.; Hyde, S. On the swelling of bicontinuous lyotropic mesophases. *J. Phys. II* **1995**, *5*, 171-190.
